# The V-line: a sonographic aid for the confirmation of pleural fluid

**DOI:** 10.1186/2036-7902-4-19

**Published:** 2012-08-24

**Authors:** Paul Atkinson, James Milne, Osama Loubani, Glenn Verheul

**Affiliations:** 1 Dalhousie Medicine New Brunswick, 100 Tucker Park Road, Saint John, NB, E2L 4L5, Canada; 2 Department of Emergency Medicine, Dalhousie University, Saint John Regional Hospital, 400 University Ave., Saint John, NB, E2L 4L2, Canada; 3 Department of Emergency Medicine, Dalhousie University, Halifax Infirmary Suite 355-1796 Summer St., Halifax, NS, B3H 3A7, Canada

## Abstract

**Background:**

Ultrasound is being used increasingly to diagnose pathological free fluid accumulation at the bedside. In addition to the detection of peritoneal and pericardial fluid, point-of-care ultrasound allows rapid bedside diagnosis of pleural fluid.

**Findings:**

In this short report, we describe the sonographic observation of the vertebral or ‘V-line’ to help confirm the presence of pleural fluid in the supine patient. The V-line sign is a result of the fluid acting as an acoustic window to enable visualization of vertebral bodies and posterior thoracic wall, thus confirming the presence of pleural fluid.

**Conclusions:**

The V-line is a useful sonographic sign to aid the diagnosis of pleural free fluid.

## Findings

### Introduction and background

The use of ultrasound for the detection of pleural fluid is reliable when correctly identified [1]. Pleural free fluid is common in patients presenting to hospital with respiratory symptoms. In one study of 880 patients presenting to emergency departments in North America and Europe with a chief complaint of shortness of breath, 17% were found to have pleural effusions [2]. As many as 62% of patients requiring admission to medical intensive care units (ICU) have pleural effusions [3], and between 10% to 20% [4-10] of patients presenting with thoracic trauma have hemothorax. Abnormal accumulation of fluid in the pleural cavity occurs when there is an imbalance between production and absorption, resulting from a variety of disease processes including, but not limited to, malignancy, heart failure, pneumonia, empyema, and traumatic bleeding. Fluid accumulation in the pleural cavity ultimately leads to lung compression and abnormalities in oxygenation or ventilation. The presence of pleural fluid is typically diagnosed through physical examination and plain radiography. Classic physical exam findings of pleural fluid have extremely poor sensitivities and specificities for the diagnosis of pleural fluid and should not be relied on for diagnosis [11].

Chest X-ray (CXR), the most commonly used radiographic modality to detect pleural effusion, is able to detect only relatively large effusions. Studies have shown that over 175 mL of fluid is required to cause blunting of the costophrenic angles in an upright CXR [12]. Supine CXR, used in critically ill and trauma patients, is even poorer at detecting pleural effusions, being able to detect 175 to 525 mL of pleural fluid [13].

Clinically, the sensitivity and specificity of CXR for pleural fluid diagnosis are relatively poor. In ICU patients with co-existing lung pathology (which represents the majority of critically ill patients), supine CXRs have a sensitivity of 39% and specificity of 85% for detection of pleural fluid [14].

The significance of pleural fluid to the patient’s presentation is not always clear, and so early detection of pleural fluid is important to guide the decision for drainage, either for diagnostic or therapeutic purposes. The use of ultrasound for the detection of pleural fluid was first described in 1967 [15]. Since that time, ultrasound has been shown to be extremely sensitive for the detection of pleural fluid, with the ability to detect as little as 20 mL [7,16].

The clinical sensitivity and specificity of point-of-care ultrasound (PoCUS) for the detection of pleural fluid have also been demonstrated. In the thoracic trauma population, PoCUS has consistently been shown to have a sensitivity between 92% to 100% and a specificity nearing 100% for hemothorax [4-10]. In the ICU population, PoCUS has demonstrated a sensitivity of 92% and specificity of 93% for the detection of pleural fluid [14], even in the presence of severe pulmonary pathology.

In addition to an excellent sensitivity and specificity for detection of pleural fluid, PoCUS drastically reduces the time to clinical diagnosis when compared to CXR. In the trauma population, PoCUS has been shown to provide a diagnosis of hemothorax within 1 min compared to 15 min by CXR [4,6]. When pleural fluid is present, an anechoic (dark) area is seen cephalad to (above) the diaphragm.

While not yet fully accepted as standard of care by some physicians, lung ultrasound is becoming established as a beneficial tool to diagnose a variety of serious respiratory conditions including pneumothorax, pulmonary edema (alveolar interstitial syndrome), and consolidation, as well as pleural effusions [17-21].

Each of the above conditions has a distinct set of sonographic findings related to the underlying pathology. By international convention, many of these findings have been named by their ‘line pattern’ [22]. A summary of some of the ultrasound findings described by their line pattern name, related pathology, and description of sonographic findings are summarized in Table 1.

**Table 1:** Line patterns seen in lung ultrasound and their corresponding clinical significance

**Line pattern**	**Clinical significance**
A lines	A lines are ultrasound artifacts that are not related to any pathology.
	These lines are horizontal in the ultrasound field and regularly spaced. They represent the ultrasound waves reflecting off of the pleural lining rather than returning directly back to the probe. This finding can be seen in normal lungs.
B lines	B lines are pathologic findings related to alveolar interstitial syndrome.
	These lines are vertical and narrow, which project from the pleural lining to the edge of the ultrasound field. They are hyper-echogenic, distinct, and resemble either rays of sun shining through clouds or ‘comet tails’. When there are multiple B lines in the image, they are described as ‘lung rockets’.
Z lines	These can be seen in healthy patients as well as in those with a pneumothorax.
	They resemble B lines but are shorter, broader, and not as clearly defined. They do not project to the edge of the ultrasound window but still arise from the pleural line.
E lines	Seen in subcutaneous emphysema or in the presence of echogenic foreign bodies.
	These can be best described as comet tail artifacts that are superficial to the pleural lining.
V line	Seen in supine patients in the presence of pleural fluid.
	The posterior thoracic cage (vertebral bodies and posterior ribs) is seen as an echogenic line extending cephalad to the diaphragm due to transmission of ultrasound waves through fluid to the posterior thoracic cavity.

In this descriptive paper, we describe the V line, a sign that we believe may help the clinician sonographer detect the presence of plural fluid in the supine patient and may also aid the less experienced sonographer to differentiate between the black ‘shadow’ or ‘curtain’ created by normal lung and the anechoic area that represents free pleural fluid. Written informed consent was obtained from the patients for publication of this report and any accompanying images.

### The V line

**Figure 1:**
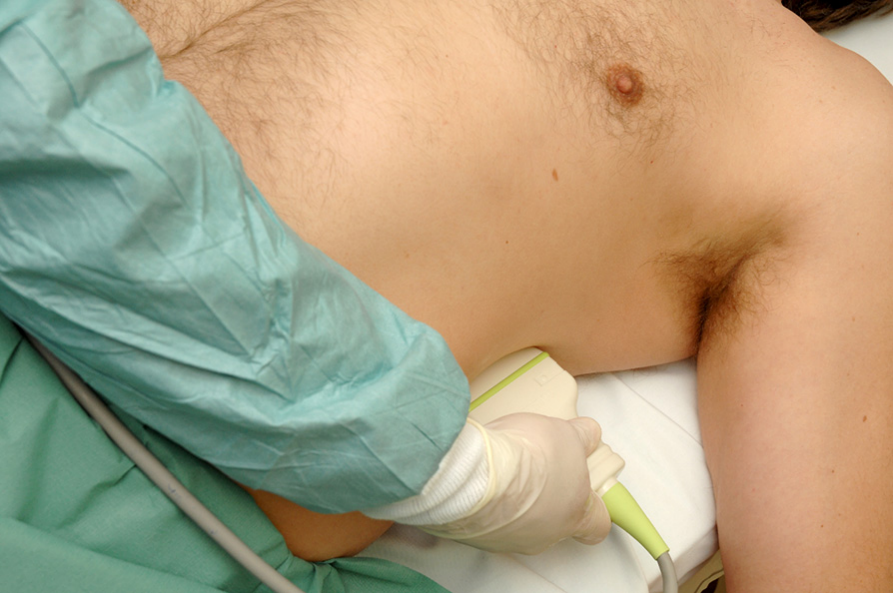
Placement of US probe for V-line visualization (left lung base).

When scanning a typical patient in a supine position using a low frequency sector probe in the coronal/longitudinal plane, laterally on the torso at the level of the diaphragm (Figure 1), one is able to clearly see the typical sonographic pattern of the vertebral bodies caudal to the diaphragm. This series of intermittent echogenic foci with acoustic shadows is visible because sound waves are transmitted to the vertebral bodies by the acoustic windows of the liver (or spleen) and kidney.

**Figure 2:**
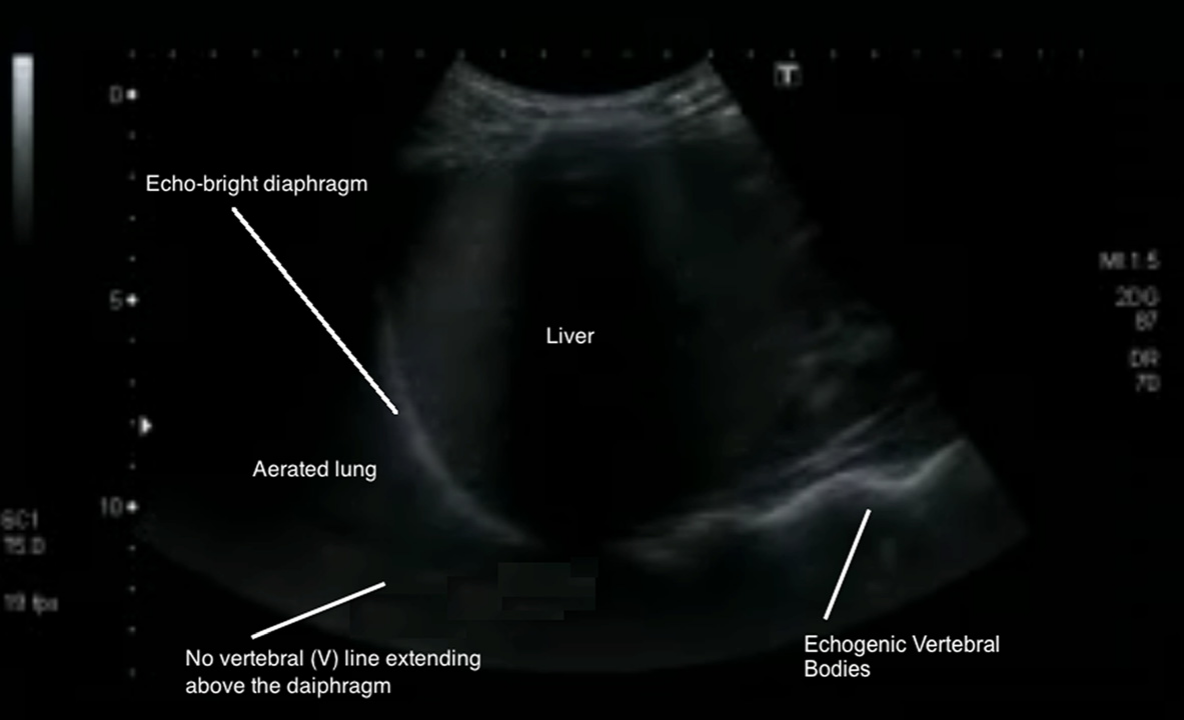
Lung ultrasound with no V line extending above the diaphragm (right lung base).

**Figure 3:**
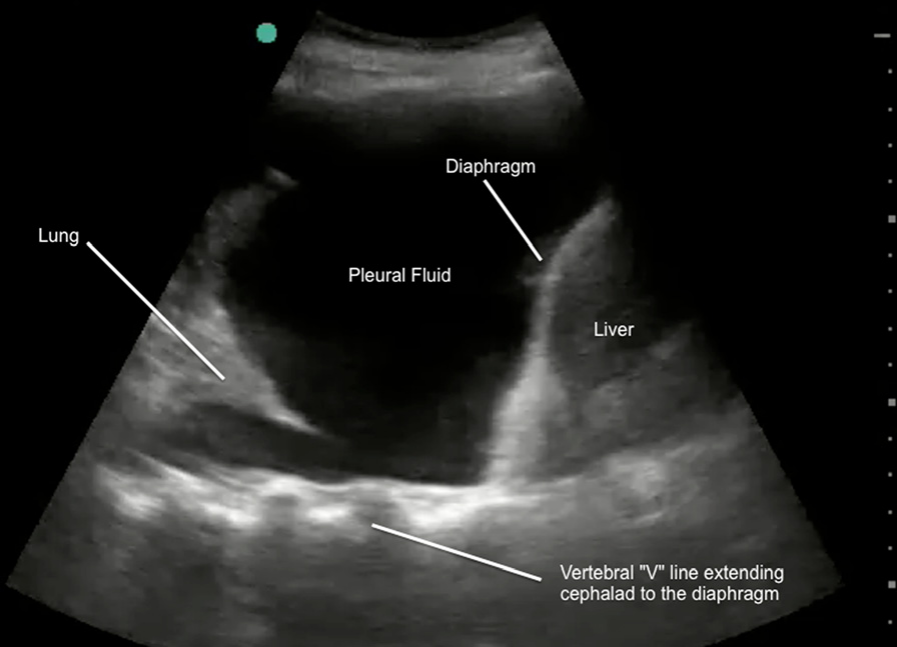
**Lung ultrasound demonstrating V line (echogenic posterior line extending above the diaphragm).** This confirms pleural fluid (right lung base).

Above (cephalad) to the diaphragm, the vertebral line ends abruptly as aerated lung does not permit transmission of sound waves to the posterior thoracic structures including the vertebral bodies and proximal posterior ribs. Instead, a black acoustic shadow or ‘lung curtain’ is seen, where no posterior structures can be visualized (Figure 2). However, the presence of even a small amount of pleural fluid in the costophrenic angle acts as an acoustic window, allowing visualization of the vertebral (V) line above the diaphragm (Figure 3).

This V-line sign can help to differentiate between fluid and air as the cause of the dark echo-poor area seen above the diaphragm with normal lung and with pleural effusions. The V line should be used in conjunction with previously described sonographic signs of pleural fluid such as, the sinusoid sign, representing the inspiratory movement of lung line toward pleural line; clearer visualization of the diaphragm and abdominal organs during inspiration; less echogenic scatter at the diaphragm; and visualization of lung tissue ‘floating’ in the pleural fluid.

## Conclusions

The V line is a simple sonographic finding that may assist the clinician with the detection of pleural fluid. We suggest that it will complement other previously described line patterns when performing lung ultrasound for symptomatic breathless patients. It offers a simple memorable sign to assist with training, help confirm the presence of posterior pleural effusions, and differentiate anechoic fluid from the normal black lung curtain. We suggest that a prospective study of the diagnostic characteristics of this sign would be useful.

## Competing interests

The authors declare that they have no competing interests.

## Authors’ contributions

GV and PA described this previously unreported sign. PA, JM and OL performed the literature review. PA and JM wrote the initial draft of the paper and acquired the clinical images. All authors read and approved the final manuscript.
